# Implementation of Febrile Infant Management Guidelines Reduces Hospitalization

**DOI:** 10.1097/pq9.0000000000000252

**Published:** 2020-01-22

**Authors:** Lauren Z. Foster, Joshua Beiner, Carol Duh-Leong, Kira Mascho, Victoria Giordani, Michael L. Rinke, Leonardo Trasande, Ethan Wiener, Rebecca E. Rosenberg

**Affiliations:** From the * Department of Pediatrics, New York University School of Medicine, Hassenfeld Children’s Hospital at NYU Langone, New York City, N.Y.; † Section on Emergency Medicine, Department of Pediatrics, Yale School of Medicine, New Haven, Conn.; † Department of Pediatrics, Children’s Hospital at Montefiore, New York City, N.Y.; § Department of Population Health, New York University School of Medicine, Hassenfeld Children’s Hospital at NYU Langone, New York City, N.Y.; ¶ Department of Emergency Medicine, New York University School of Medicine, Hassenfeld Children’s Hospital at NYU Langone, New York City, N.Y.

## Abstract

The clinical management of well-appearing febrile infants 7−60 days of age remains variable due in part to multiple criteria differentiating the risk of a serious bacterial infection. The purpose of this quality improvement study was to standardize risk stratification in the emergency department and length of stay in the inpatient unit by implementing an evidence-based clinical practice guideline (CPG).

**Methods:**

The Model for Improvement was used to implement a CPG for the management of well-appearing febrile infants, with collaboration between pediatric emergency medicine and pediatric hospital medicine physicians. Interventions included physician education, process audit/feedback, and development of an electronic orderset. We used statistical process control charts to assess the primary aims of appropriate risk stratification and length of stay.

**Results:**

Over a 34-month period, 168 unique encounters (baseline n = 65, intervention n = 103) were included. There was strong adherence for appropriate risk stratification in both periods: the proportion of low-risk patients admitted inappropriately decreased from 14.8% to 10.8%. Among admitted high-risk patients, the mean length of stay decreased from 49.4 to 38.2 hours, sustained for 18 months.

**Conclusion:**

CPG implementation using quality improvement methodology can increase the delivery of evidence-based care for febrile infants, leading to a reduction in length of stay for high-risk infants.

## Abstract 2

Supplemental Digital Content is available in the text.

## INTRODUCTION

Fever in infants under 60 days of age is a common diagnostic scenario in emergency departments and pediatric inpatient units. This population is at risk for serious bacterial infections (SBIs) with approximately 9%−10% ultimately diagnosed with a urinary tract infection (UTI), bacteremia, or meningitis.^1–6^ A fever may be the only presenting symptom of an SBI in a well-appearing infant. Risk stratification criteria can help differentiate infants at high risk for an SBI and guide management.^7^ Because multiple risk stratification criteria are available, significant variability in practice exists regarding laboratory evaluation, antibiotic treatment, and admission.^5,8,9^ Furthermore, although there is ample evidence regarding modern microbiologic techniques and time to culture positivity,^10^ there remains variability in the duration of culture monitoring deemed sufficient before discharge.

**Fig. 1.**
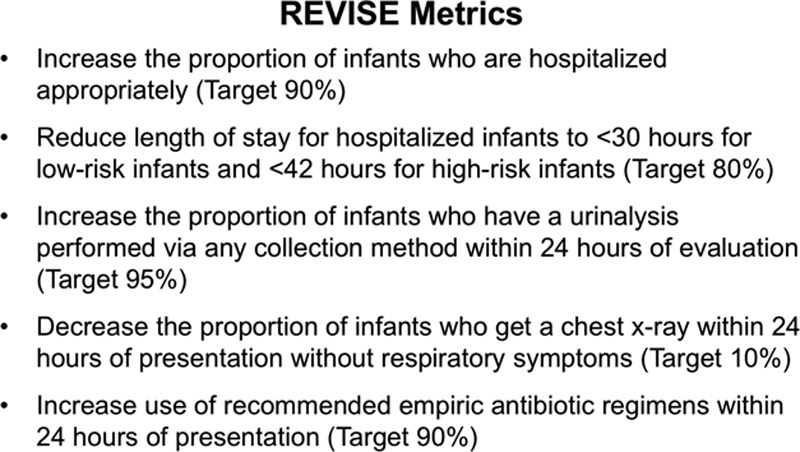
National collaborative metrics (American Academy of Pediatrics: Reducing Excessive Variability in Infant Sepsis Evaluation^13^), adapted for the local project.

This variation in the management of febrile infants with nonfocal examinations can lead to overtreatment with non–evidence-based tests, therapies, and hospitalizations, which may provide little clinical benefit and may increase infant exposure to harm and unnecessary resource utilization.^2,5,6,11^ Implementation of clinical practice guidelines (CPGs) using quality improvement (QI) methodology can decrease variation, improve quality of care, and decrease costs.^6,12^ Our hospital participated in the American Academy of Pediatrics’ Value in Inpatient Pediatrics network’s national QI collaborative, Reducing Excessive Variability in Infant Sepsis Evaluation (REVISE), which aimed to standardize care according to 5 evidence-based metrics (Fig. 1).^13^ The overall results of this collaborative are reported elsewhere.^13^

The objective of this QI study was to implement an evidence-based CPG to decrease variation in the care of well-appearing febrile infants, 7−60 days of age, by increasing adherence to appropriate risk stratification and disposition recommendations in the pediatric emergency department (PED) and decreasing the length of stay (LOS) for hospitalized infants. We planned to use continuous QI methodology to achieve and measure adherence and report here on pragmatic implementation strategies from the perspective of one hospital engaged in a multisite QI effort.

## METHODS

### Setting

We conducted this QI project at Hassenfeld Children’s Hospital at New York University (NYU) Langone Health, an urban academic children’s hospital within a hospital with 9,200 PED visits and 2,300 inpatient unit admissions annually (≈35% publicly insured). The PED is staffed by pediatric emergency medicine (PEM) and general pediatric attendings with pediatric and emergency medicine house staff. The general pediatric acute care team is primarily staffed by pediatric hospital medicine (PHM) attendings with pediatric house staff.

### Population

We included in the CPG well-appearing infants 7−60 days of age with fever ≥38.0°C at arrival evaluated in the PED. We excluded infants for the following reasons:

1. if they were “ill-appearing” at presentation as identified by key phrases indicating appearance, work of breathing, and perfusion;
2. if intensive care was required;
3. if unstable vital signs such as hypotension were present;
4. if a focal source of illness by history or physical examination (eg, cellulitis) was identified; or
5. if a comorbid condition predisposing to severe or recurrent bacterial illness, including genetic, congenital, neuromuscular, or neurodevelopmental abnormalities, was present.

### Planning the Intervention

We assembled a multidisciplinary team that included 2 PHM attendings, 2 PEM attendings, 2 pediatric house staff, and a pharmacist. We used the modified Model for Improvement with multiple Plan-Do-Study-Act cycles.^14^ As a participant in the national collaborative REVISE,^13^ we utilized similar metrics (Fig. 1) and adapted education materials, chart review tools, and algorithms to fit site-specific needs.

Our global aim was to promote standardized, evidence-based care of febrile infants 7−60 days of age in our PED and inpatient unit. Given the impact of hospitalization on patients and families, resource use, and cost, our aims emphasized (1) avoidance of low-risk admissions and (2) decreased LOS.

PED patient stratification into low- and high-risk categories was essential to determine the appropriate disposition (low risk: discharge; high risk: admit). We based this risk stratification on a patient’s age, medical history, and laboratory evaluation (inflammatory marker and urinalysis). As with the national REVISE data collection, “low-risk” infants were defined as those older than 30 days of age with no significant medical history and normal laboratory testing by Rochester criteria^15^ and no documented social concerns related to follow-up. All other infants were “high-risk.”^13^

LOS recommendations were <24 and <36 hours for low- and high-risk patients, respectively, due to evidence that 95% of true pathogens grow in blood cultures by 36 hours.^10^ Adding a 6-hour margin for the initial culture collection and logistical issues surrounding discharge, we used 30- and 42-hour LOS goals for low- and high-risk patients, respectively. Infants with an SBI, defined as a blood, urine, or cerebrospinal fluid (CSF) culture positive for a pathogenic organism,^13^ were excluded from LOS recommendations. We considered infants who returned to the PED or were readmitted with a new diagnosis of bacteremia, UTI, or meningitis within 7 days of the initial presentation to have a missed SBI.

Therefore, our site-specific improvement aims were to (1) improve PED adherence to a standardized risk stratification and disposition guideline to decrease low-risk admissions from 15% to <10% and (2) increase inpatient adherence to LOS recommendations to discharge high-risk patients within 42 hours from 31% to 80%. The balancing measure was to maintain a missed SBI rate of <2%. We aligned these goals with the national collaborative metrics.^13^

**Fig. 2.**
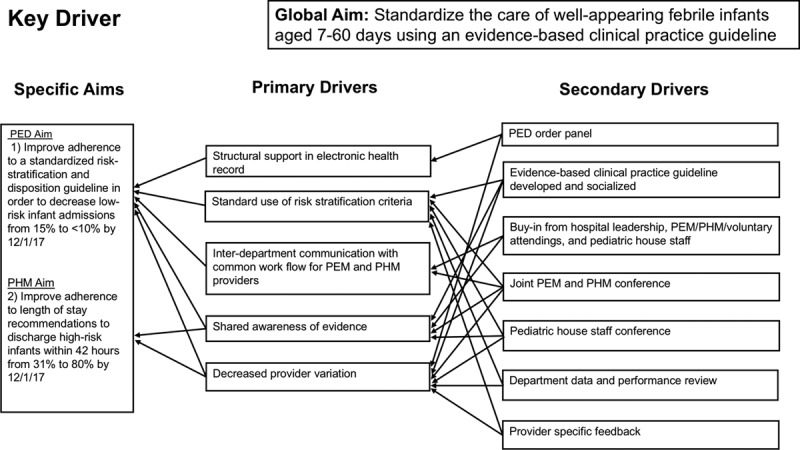
Key driver diagram outlining the specific aims, primary drivers, and secondary drivers for the project goal of standardizing the care of well-appearing febrile infants 7−60 days of age using a clinical practice guideline.

The team identified several key drivers, including a shared awareness and consensus on a standardized approach to the care of febrile infants, physician knowledge of evidence-based management, and electronic ordering support (Fig. 2). These drivers resulted from discussions with PEM and PHM physicians and department administration, pediatric infectious disease and critical care leadership, pharmacists, informaticists, and community pediatricians. The interventions targeting these key drivers included collaborative development of a site-specific evidence-based CPG, physician education (PEM, PHM, and pediatric house staff), audit and feedback systems, and creation of a PED febrile infant order panel. The team met monthly throughout the intervention period (December 2016 to June 2018) to review data, assess the effects of the interventions, and plan subsequent Plan-Do-Study-Act cycles.

### Interventions

1. Socializing the intervention (December 2016): The team introduced the national project REVISE and its aims^13^ to PEM and PHM faculty at individual division and joint meetings. We shared local baseline adherence data, specifically emphasizing appropriate risk stratification (primary PED aim) and LOS (primary PHM aim).
2. CPG development (January 2017 to March 2017): We adapted our site-specific CPG from the REVISE materials^13^ and the Rochester criteria^15^ for risk stratification (see **Supplemental Digital Content** at ***http://links.lww.com/PQ9/A156*** for Figs. A and B). The PED portion of the CPG outlined recommended laboratory tests for infants based on age and bacterial and herpes-simplex virus (HSV) infection risk factors, with a corresponding risk stratification and disposition recommendation. The inpatient CPG provided recommendations for length of culture monitoring before discharge, with considerations for an earlier discharge based on viral testing.

There were several differences in our CPG from the national collaborative in areas of clinical controversy or site-specific needs. We based these changes on review of the literature, feedback from stakeholders, and group consensus. We recommended a routine lumbar puncture on all infants 28 days of age and younger with subsequent determination of empiric antibiotic administration based on risk instead of using the clinical decision to administer antibiotics to guide the performance of a lumbar puncture. We revised our CPG to include a rapid CSF pathogen panel newly available at our institution, given physician feedback on the utility of this test and process issues surrounding stepwise ordering. Our CPG also included additional HSV risk factors as a result of our unique local population and epidemiology.^16^

3. Spread of guidelines (January 2017 to March 2017): We provided initial education on the CPG at a joint PHM and PEM conference, with a similar conference held for the pediatric house staff. These conferences reviewed the goal of increasing adherence to evidence-based recommendations, the underlying evidence behind the CPG, and the specific project aims with our baseline data. Outreach to voluntary and referring pediatricians occurred by email with the support of the chief of service and PEM chief. The CPG was available on the Children’s Services website and house staff resource website. We also posted it in the PEM physician space and acute care house staff workroom. The national collaborative’s mobile application was also promoted among house staff and faculty.^13^
4. Continuous QI (December 2016 to June 2018): We reviewed and discussed data at monthly division meetings for PHM and PEM and quarterly with pediatric house staff. After team discussion of any deviations from the CPG identified on case review, the respective specialty would respectfully reach out to their faculty member via email with an explanation of the missed metric and a request for feedback regarding barriers to CPG implementation.
5. PED order panel (October 2017): The team developed and implemented an electronic health record order panel to provide structural support for the initial ordering process in the PED. This panel included standard laboratory tests and empiric antimicrobial coverage, reviewed by the pediatric clinical pharmacist and pediatric infectious disease leaders. The intent was to ensure that all necessary and recommended tests were ordered and to reduce those tests for which there is no broad recommendation to perform routinely. The team provided ongoing reminders and education to PEM faculty and fellows regarding the order panel.

### Data Collection

We collected 15 months of baseline data (September 1, 2015, to November 30, 2016) and 19 months of prospective intervention phase data (December 1, 2016, to June 23, 2018), the latter monthly. A report was generated through the electronic health record with a list of patients 7−60 days of age seen in the PED. We used any admission diagnosis, the reason for the visit, or principal problem that included “fever,” “urinary tract infection,” “meningitis,” or “bacteremia” to identify possible patients. Using a chart review tool, we evaluated each potential case to determine if the patient met inclusion criteria and to extract data. Each case underwent a secondary review by the principal investigator (L.Z.F.). Chart reviewers included physician project team members who were trained in person by the principal investigator and received ongoing feedback based on principal investigator secondary review. Data were deidentified and imported into an Excel spreadsheet.

The REVISE national study was approved by the American Academy of Pediatrics institutional review board (IRB).^13^ The NYU School of Medicine IRB determined our project to be QI and not human subjects research. Therefore, review and approval by our local IRB were not required.

### Analysis

We used statistical process control charts for analysis of QI metrics, employing established rules for identifying special cause variation.^17,18^ Although the CPG listed 7−28 and 29−60 days as the cut-points, we categorized data by 30 days and younger and 31−60 days due to equipoise around the 28−30 days window as per the national project.^13^

Adherence to the PED risk stratification bundle was defined by appropriate laboratory testing (inflammatory marker and urinalysis sent) and disposition (discharge for low-risk patients). We evaluated overall adherence to the bundle with a run chart and a pre-/postanalysis using the Fisher’s exact text. Given that our baseline data showed high adherence to the risk stratification laboratory testing, we focused on the disposition portion for our aim, specifically the avoidance of low-risk admissions. Low-risk admissions were analyzed with a G chart due to their low frequency. A pre-/postanalysis of the proportion of admitted low-risk infants was performed using the Fisher’s exact text.

For inpatient process adherence, we examined LOS in hours for high-risk infants using an XmR chart. We excluded patients from the LOS analysis if they were diagnosed with an SBI. A pre-/postanalysis of the median LOS in hours and days for high-risk patients was performed using the Mann–Whitney–Wilcoxon test. The proportion of cases adherent to LOS goals for low- and high-risk infants was examined with run charts and pre-/postanalyses using the Fisher’s exact text.

The balancing measure of a missed SBI would be a rare event since the reported rate of low-risk patients with an SBI using various risk stratification criteria is 0.67%−2.71%.^7^

## RESULTS

Over a 34-month period, 168 unique encounters (baseline n = 65, intervention n = 103) were included. The proportions of infants 30 days of age and younger (35.4% and 34.0%; *P* = 0.85) and stratified as high risk (58.3% and 64.1%; *P* = 0.47) were similar between baseline and intervention groups. There was no significant difference between groups regarding sex or insurance (Table 1).

**Table 1.**
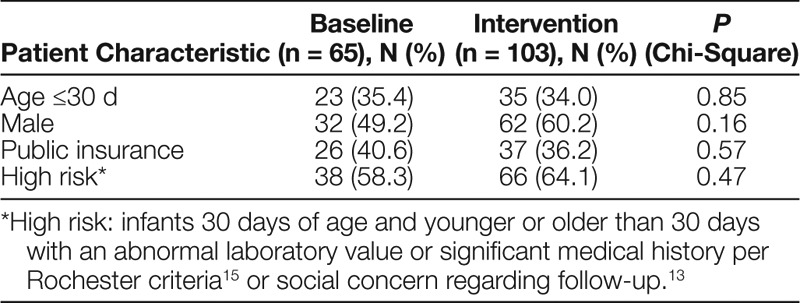
Demographics of Well-Appearing Febrile Infants 7−60 Days of Age by Study Phase, n = 168

### Process Aim 1: PED Adherence to Risk Stratification and Disposition

**Fig. 3.**
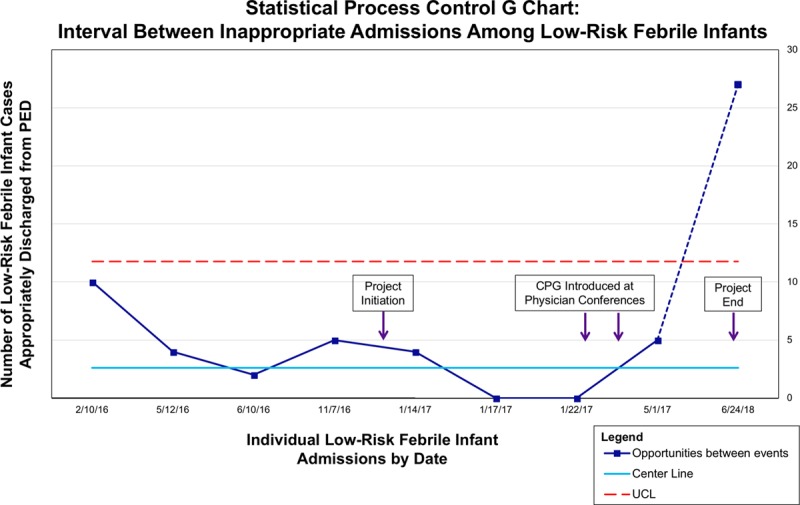
Statistical process control G chart of the interval between inappropriate admissions among low-risk febrile infants. Special cause variation is seen within 6 months of project initiation and implementation of the clinical practice guideline (CPG). No inappropriate low-risk admissions occurred during the final 13 months of data collection. UCL, upper control limit.

Adherence to the risk stratification and disposition bundle in the PED did not significantly change after CPG implementation. Baseline compliance was 92.3% (60/65) compared with 91.3% (94/103) in the intervention phase (*P* = 1). The proportion of low-risk patients admitted, although meeting guideline criteria for discharge, decreased from a baseline of 14.8% (4/27) to 10.8% (4/37) during the intervention period. This difference was not statistically significant (*P* = 0.71). A G chart of low-risk admissions demonstrated special cause variation within 6 months of project initiation (Fig. 3). There were no low-risk admissions during the final 13 months of data collection. The rest of the deviations from the risk stratification bundle were due to a lack of urinalysis. This deficiency occurred in 1.5% (1/65) baseline and 4.9% (5/103) intervention period encounters and was not significantly different (*P* = 0.41). In all cases, a urinalysis was ordered but not resulted due to an insufficient urine volume sent to the laboratory, and all infants were admitted based on other high-risk factors (age, medical history, or inflammatory marker).

### Process Aim 2: Inpatient Adherence to LOS

**Fig. 4.**
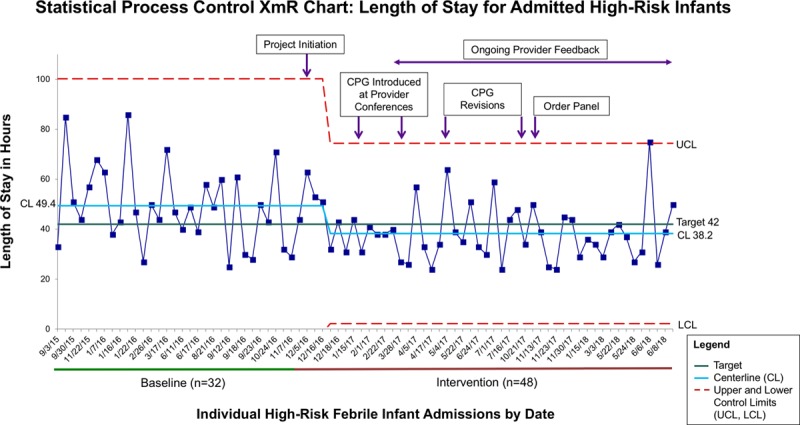
Statistical process control XmR chart of the length of stay of admitted high-risk infants with each point representing an individual patient. CL shift occurred after project initiation with a decrease in length of stay from 49.4 to 38.2 hours, meeting the target of <42 hours. The timing of various interventions, including clinical practice guideline (CPG) development, physician education, feedback, and order panel launch, is outlined. Special cause at June 6, 2018, was a prolonged stay due to attending physician concern for bacterial meningitis in consultation with pediatric infectious disease. CL indicates centerline.

The XmR chart for LOS for high-risk admitted patients demonstrated a centerline shift and special cause variation soon after project initiation in December 2016 (Fig. 4). LOS decreased from 49.4 to 38.2 hours shortly after the project began with PHM faculty engagement. We sustained this improvement for 18 months. In pre-/postcomparison, the median LOS for high-risk patients decreased from 47 hours [interquartile range (IQR), 39–59] to 39 hours (IQR, 31–44), *P* = 0.02, whereas the median inpatient days decreased from 3 (IQR, 2–3) to 2 (IQR, 2–3), *P* = 0.004. The overall adherence to the <42-hour LOS recommendations for high-risk infants increased from a baseline of 31.3% (10/32) to 66.7% (32/48) during the intervention phase (*P* = 0.003). Of the low-risk infants admitted, only 1 of 4 was discharged by 30 hours during the baseline period, whereas all 4 met this goal in the intervention phase.

### Balancing Measure

We identified no patients with a missed SBI during the baseline period. There was one patient with a missed SBI in the intervention period (1/103 = 0.97%) within the goal rate of <2%. This patient was older than 30 days of age and high risk by guideline stratification due to abnormal laboratory values, discharged from the PED, and recalled to the PED for outpatient treatment of a UTI. There were no readmissions within 7 days of the initial presentation in either period.

## DISCUSSION

We demonstrated the efficacy of a multidisciplinary approach to decreasing unnecessary hospitalization for well-appearing febrile infants 7−60 days of age at a children’s hospital within a hospital, using the Model for Improvement to implement a CPG as part of a multisite QI collaborative. Baseline data reflected a high adherence to risk stratification and disposition recommendations for affected infants at our institution, and there were no inappropriate low-risk admissions for 13 months before study completion. We achieved our aim of a shorter LOS for admitted high-risk infants by 11 hours, with sustained results over 18 months. Key drivers to achieve these aims were an interdepartmental collaboration with leadership support, continual evidence-based education, and tailoring of recommendations to fit site-specific needs.

Management of the young well-appearing febrile infant has been an area of clinical controversy, given the lack of national guidelines and the existence of multiple risk stratification criteria.^2,19^ Physician variability in the adoption of new evidence-based practices exists, compounding the known gap between knowledge development and translation to clinical practice.^5,8,9,20^ A CPG provides physician support for clinical decision-making and can be an effective way to decrease variation in care, improve patient outcomes, and decrease costs.^6,21^ However, physicians may be hesitant to embrace national recommendations or participate in multisite initiatives if the approach differs greatly from their own experience and practice.^22^

We successfully implemented and sustained our CPG (Supplemental Digital Content at ***http://links.lww.com/PQ9/A156*** for Figs. A and B), revised from a national initiative,^13^ and achieved our primary measures. We detail here specific barriers and facilitators for single institutions seeking to accomplish these goals. Our primary barrier was variability in physician practice stemming from a lack of consistent education on this topic and diverse experience. Collaboration between PEM and PHM physicians with an interdepartmental commitment to evidence-based medicine was key to standardizing our management approach for febrile infants. Gaining perspectives from all stakeholders was crucial in CPG creation, and adoption of the CPG into the practice culture was the result of champions from both PEM and PHM, including section leaders. Ongoing education with continual general and specific feedback for pediatric faculty and house staff reinforced the core CPG concepts. The high frequency of the clinical scenario may have also helped attendings and house staff use, teach, and promote CPG use.

Risk stratification is essential to the management of febrile infants because it allows for the identification of infants at higher risk for an SBI while reducing unnecessary exposures for low-risk infants.^4,7^ Using common risk stratification criteria, instead of physician-dependent use of various criteria, may have helped avoid low-risk admissions. Because deviations to risk stratification were already infrequent at baseline, a CPG may be of higher yield in reducing hospitalization for this low-risk population at other sites. This shared mental model also promoted consistent expectations between PEM and PHM physicians regarding the admission decision.

With advanced technologies in viral testing and monitoring of cultures, there is increasing evidence to support a shorter LOS of 24−36 hours for young febrile infants as opposed to the traditional 48-hour observation period.^1,6,23–26^ Decreasing unnecessary or prolonged hospitalizations can positively affect resource utilization and cost,^6^ decrease complications and iatrogenic harms,^11,27,28^ and alleviate parental psychologic stress^29,30^ and financial hardships.^31^ This goal was achievable and sustainable, and our success is relevant for individual sites that are committed to improvement.

There will continue to be some variability in LOS based on the timing of culture acquisition and inpatient admission, medical complications (eg, dehydration), and social barriers (eg, transportation). Another barrier to timely discharge was physician discomfort with an incomplete evaluation, particularly lack of or partial CSF studies, because these scenarios remain subject to individual physician judgment and are not part of the CPG.

Our CPG, while following evidence-based recommendations, incorporated site-specific considerations which addressed additional points of physician practice variability. We established a consensus for performing lumbar punctures for less than or equal to 28-day-old infants, an area of ambiguity in the literature, and incorporated newly available CSF pathogen testing. For our local population, it was also important to include HSV risk factors and indications for empiric antiviral treatment. This modification demonstrates how a national collaborative’s algorithms can be adapted to serve individual sites best and achieve the same aims.

### Limitations

Generalizability is limited as we performed this study at a single academic children’s hospital within a hospital with small sample size. A rapid CSF pathogen panel was introduced in the institution during our intervention phase and may have impacted risk stratification and LOS. Correlation of viral testing and LOS was not analyzed because it was not a focus of the study.

## CONCLUSIONS

We demonstrated sustained significant adherence to evidence-based care for young febrile infants in both the PED and inpatient units of a children’s hospital within a hospital using a CPG. This improvement was associated with decreased hospitalization. Key drivers were an interdepartmental collaboration, continual education with consistent audit and feedback at the group and individual level, a focus on site-specific needs, and support from a national collaborative. These results demonstrate the key interventions on a local level that can lead to hospitalization-related resource and cost savings on a larger scale given the prevalence of this clinical population and simultaneous focus on this topic in a national network.^12,13^

## ACKNOWLEDGMENTS

The authors thank Robert Witcher, PharmD, BCCCP, Chelsea Kadish, MD, and the faculty from the Section of Pediatric Hospital Medicine and the Division of Pediatric Emergency Medicine at NYU Langone for assistance with the study.

## Disclosure:

The authors have no financial interest to declare in relation to the content of this article.

## Supplementary Material

SUPPLEMENTARY MATERIAL
