# Supplementary material for: Implementation of Febrile Infant Management Guidelines Reduces Hospitalization
Source: Pediatr Qual Saf. 2020 Jan 22;5(1):e252. doi: 10.1097/pq9.0000000000000252 (PMC7056289; doi:10.1097/pq9.0000000000000252)
Supplement: SUPPLEMENTARY MATERIAL [file pqs-5-e252-s001.pdf]

SDC, Figure A. Clinical Practice Guideline for the Evaluation and Treatment of Febrile Infants Without a Source aged 7-28 DOL*

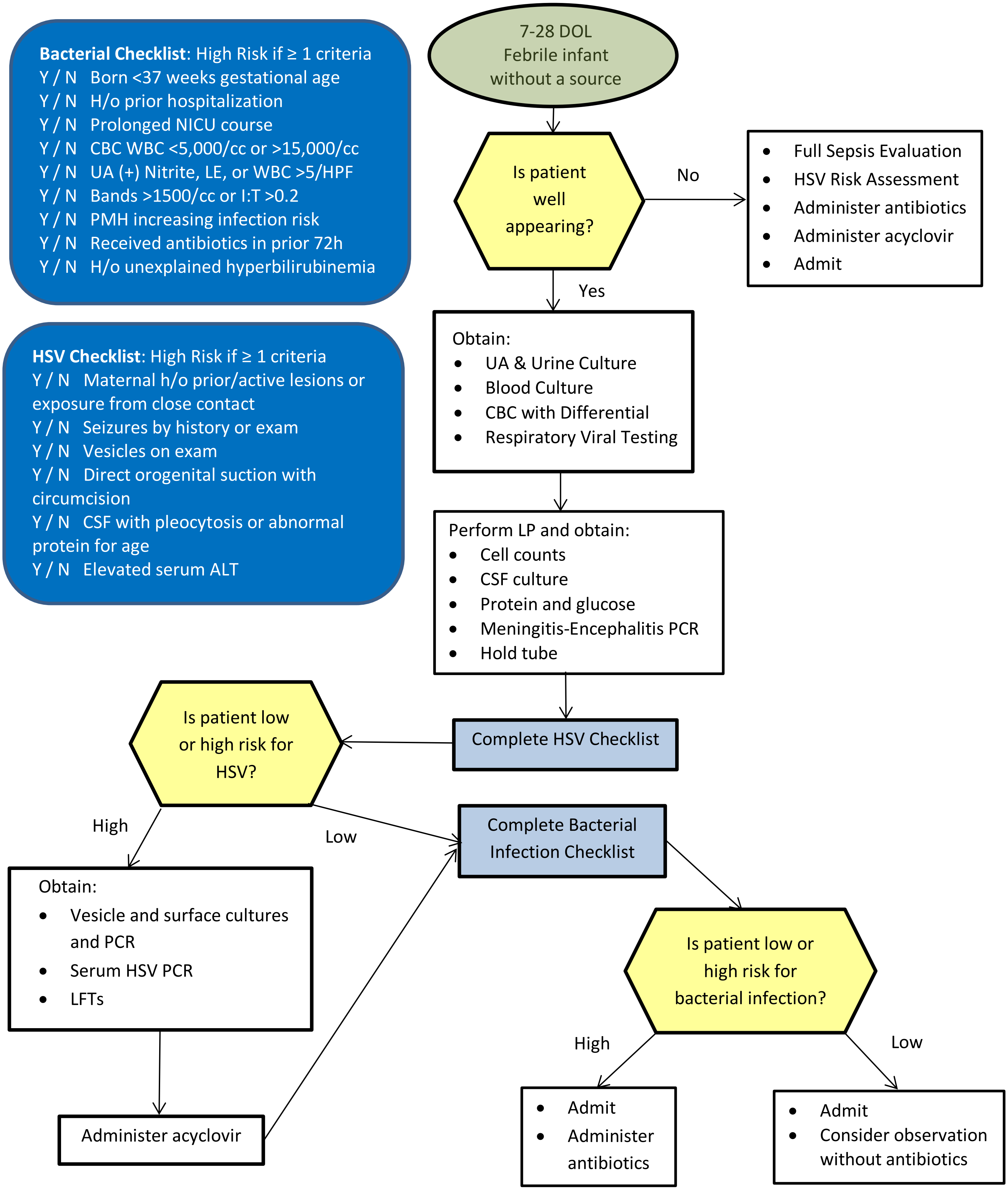

HASSENFELD CHILDREN'S HOSPITAL AT NYU LANGONE

* These are the preferred pathways that limit unnecessary interventions and exposures. Ultimately, clinical judgment and joint decision-making may dictate final work up, treatment, and disposition.

10/30/2017

SDC, Figure A. Clinical Practice Guideline for the Evaluation and Treatment of Febrile Infants Without a Source aged 29-60 DOL*

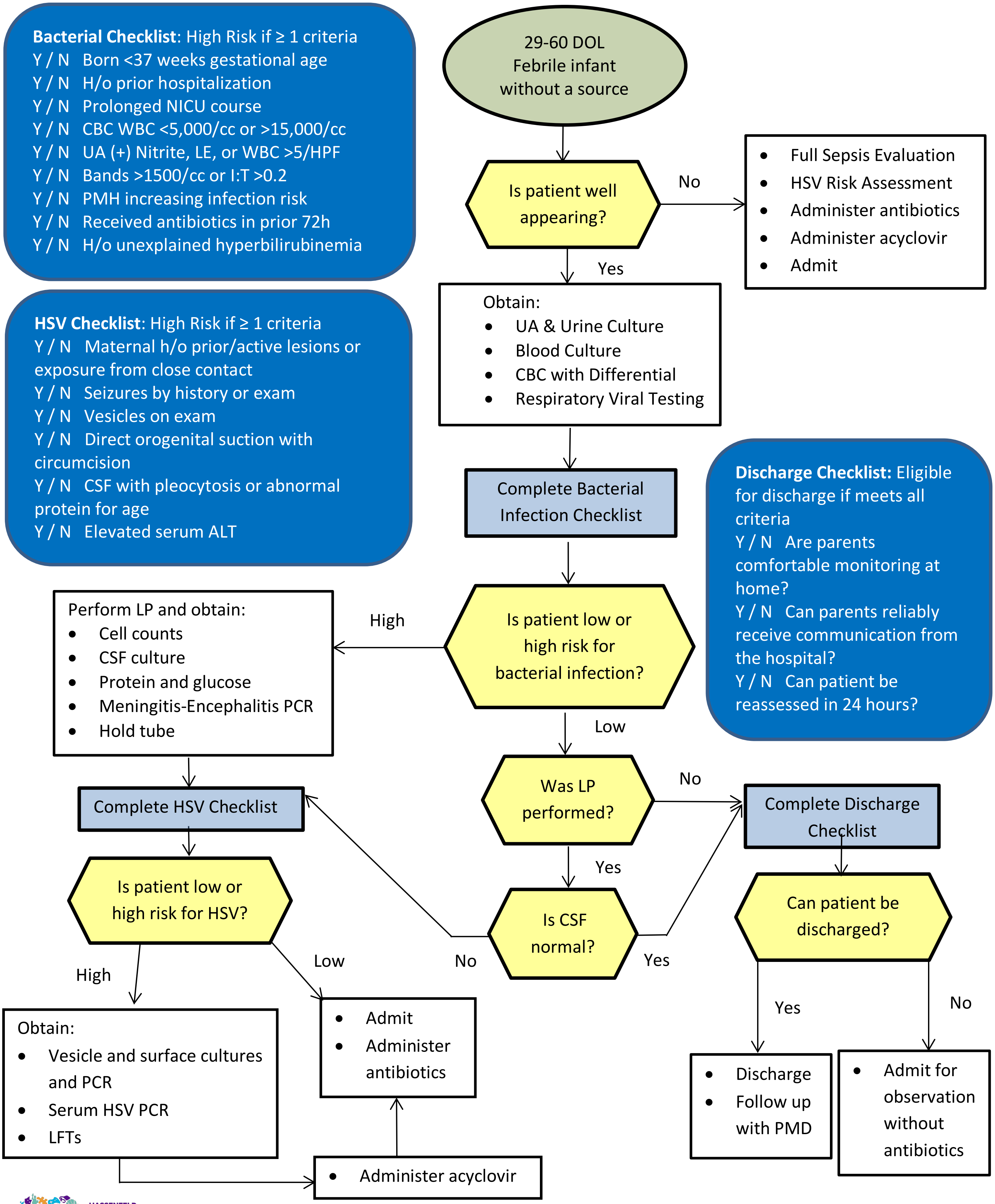

* These are the preferred pathways that limit unnecessary interventions and exposures. Ultimately, clinical judgment and joint decision-making may dictate final work up, treatment, and disposition.

10/30/2017
