# Supplementary material for: Implementation of Febrile Infant Management Guidelines Reduces Hospitalization
Source: Pediatr Qual Saf. 2020 Jan 22;5(1):e252. doi: 10.1097/pq9.0000000000000252 (PMC7056289; doi:10.1097/pq9.0000000000000252)
Supplement: SUPPLEMENTARY MATERIAL [file pqs-5-e252-s002.pdf]

SDC, Figure B. Clinical Practice Guideline for the Inpatient Treatment and Disposition of Febrile Infants Without a Source aged 7-28 DOL*

Bacterial Checklist: High Risk if ≥ 1 criteria

- Y / N Born <37 weeks gestational age
- Y / N H/o prior hospitalization
- Y / N Prolonged NICU course
- Y / N CBC WBC <5,000/cc or >15,000/cc
- Y / N UA (+) Nitrite, LE, or WBC >5/HPF
- Y / N Bands >1500/cc or I:T >0.2
- Y / N PMH increasing infection risk
- Y / N Received antibiotics in prior 72h
- Y / N H/o unexplained hyperbilirubinemia

7-28 DOL Febrile infant without a source

→ Complete Bacterial Infection Checklist

→ Is patient low or high risk for bacterial infection?

High

- Admit
- Administer antibiotics

→

- Discharge after 36 hours of negative cultures
- If positive viral testing with correlating symptoms and history, consider discharge after 24 hours of negative cultures

Low

- Admit
- Consider observation without antibiotics

→

- Discharge after 24-36 hours of negative cultures
- If positive viral testing with correlating symptoms and history, consider discharge after 24 hours of negative cultures

Preferred empiric antibiotic regimen: Cefotaxime +/- Ampicillin

HSV High Risk

- Treat with acyclovir as clinically appropriate until confirmatory labs have resulted

If any bacterial cultures or HSV testing grow a pathogen or if CSF pleocytosis is present, treat appropriately and exit pathway.

HASSENFELD CHILDREN'S HOSPITAL AT NYU LANGONE

* These are the preferred pathways that limit unnecessary interventions and exposures. Ultimately, clinical judgment and joint decision-making may dictate final work up, treatment, and disposition.

10/30/2017

SDC, Figure B. Clinical Practice Guideline for the Inpatient Treatment and Disposition of Febrile Infants Without a Source aged 29-60 DOL*

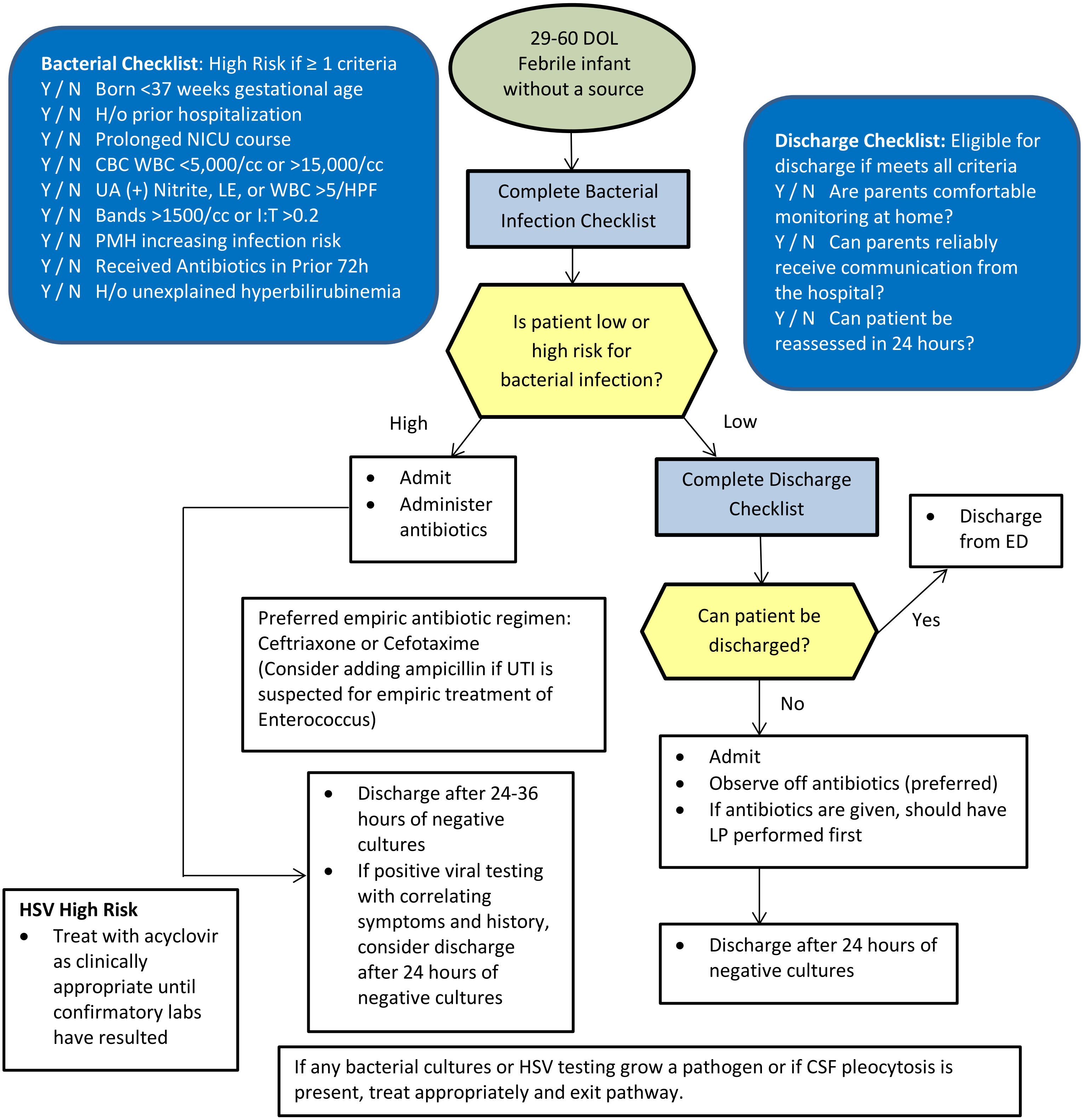

HASSENFELD CHILDREN'S HOSPITAL AT NYU LANGONE

* These are the preferred pathways that limit unnecessary interventions and exposures. Ultimately, clinical judgment and joint decision-making may dictate final work up, treatment, and disposition.

10/30/2017
